# Editorial: Innate immune dysregulation: a driving force of autoimmunity and chronic inflammation

**DOI:** 10.3389/fimmu.2025.1632416

**Published:** 2025-06-09

**Authors:** Reza Akbarzadeh, Jens Y. Humrich, Tamás Németh, Kyle T. Amber

**Affiliations:** ^1^ Department of Rheumatology and Clinical Immunology, University of Lübeck, Lübeck, Germany; ^2^ Department of Physiology, Semmelweis University School of Medicine, Budapest, Hungary; ^3^ MTA-SE “Lendület” Translational Rheumatology Research Group, Hungarian Academy of Sciences and Semmelweis University, Budapest, Hungary; ^4^ Department of Rheumatology and Immunology, Semmelweis University, Budapest, Hungary; ^5^ Department of Internal Medicine and Oncology, Semmelweis University, Budapest, Hungary; ^6^ Department of Dermatology, Rush University Medical Center, Chicago, IL, United States

**Keywords:** innate immunity, immune dysregulation, autoimmunity, chronic inflammation, inflammatory mechanisms

## 1 Introduction

Innate immunity plays a critical role in protecting the host against infections, tumors, and tissue damage by initiating inflammatory responses, recruiting immune cells, and orchestrating the production of both pro- and anti-inflammatory mediators (1). Traditionally considered the first line of defense, emerging evidence reveals that innate immunity operates through far more complex mechanisms. It not only responds to a wide array of pathogens but also engages in intricate crosstalk with the adaptive immune system (2). Importantly, its dysregulation is increasingly linked to various pathological conditions.

**Figure 1:**
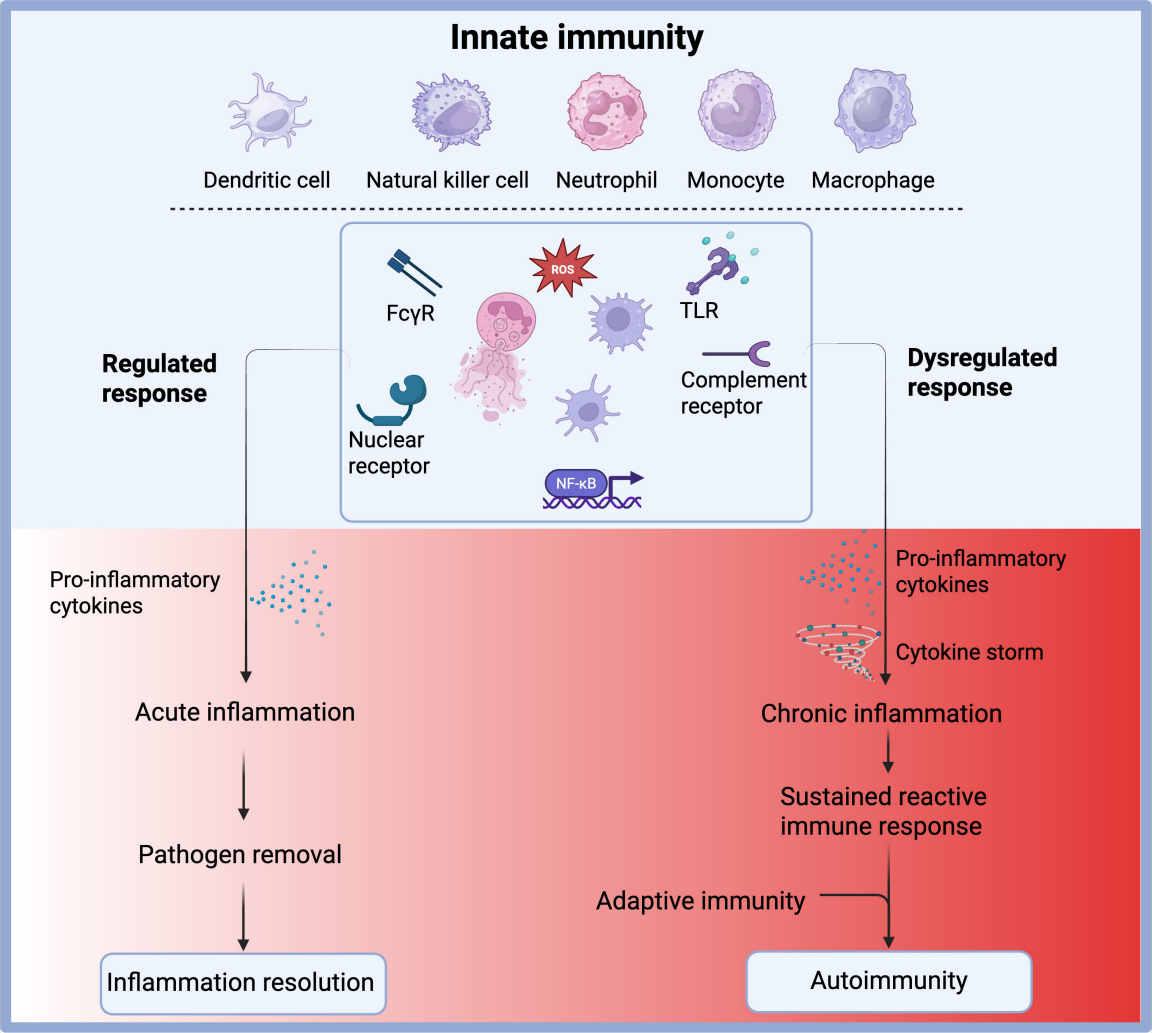
Outcomes of innate immune activation under regulated versus dysregulated conditions. Innate immune cells, including dendritic cells, natural killer (NK) cells, neutrophils, monocytes, and macrophages, respond to pathogens through receptors such as FcγR, TLRs, complement receptors, and nuclear receptors. These pathways converge on transcription factors like NF-κB and promote the production of pro-inflammatory cytokines and reactive oxygen species (ROS). Under a regulated response, this leads to acute inflammation, effective pathogen clearance, and resolution of inflammation. However, under dysregulated conditions, excessive cytokine production may trigger a cytokine storm and chronic inflammation, resulting in a sustained reactive immune state. This can aberrantly activate the adaptive immune system and contribute to the development of autoimmunity. Created with BioRender.

The initiation, activation, and resolution of innate inflammatory responses must be tightly controlled to ensure effective pathogen clearance and preservation of tissue homeostasis, while preventing excessive or prolonged inflammation. When this regulation fails, it can lead to autoinflammatory diseases and significantly contribute to chronic inflammation and autoimmune disorders (**Figure 1**) (3) such as systemic lupus erythematosus (SLE), rheumatoid arthritis (RA), and juvenile idiopathic arthritis (JIA). Although autoimmune diseases are typically characterized by a breakdown in self-tolerance associated with the adaptive immune system, the innate immune system plays a fundamental role in their onset, progression, and chronicity.

Over recent decades, autoimmune and chronic inflammatory diseases have become a growing clinical challenge, marked by rising incidence and a lack of effective, long-term treatments (4, 5). Despite advances in understanding their underlying mechanisms, current therapies largely rely on non-specific immunosuppressants, which are limited by suboptimal efficacy and potential safety concerns. These limitations often result in high disease burden, morbidity, and, in many cases, mortality. There is, therefore, an urgent unmet medical need for precisely modulating innate immune responses that reduce pathological inflammation without compromising overall immune function. Given the significant role of innate immune dysregulation in driving autoimmune and chronic inflammatory conditions, fostering scientific insights into the mechanisms by which innate immunity contributes to autoimmunity and chronic inflammation holds promise for identifying new therapeutic targets.

## 2 Molecular mechanisms of inflammation regulation

While the activation of immune responses is necessary to defend the body, unchecked or prolonged inflammation can lead to tissue damage and chronic disease. A central component of this regulation is the transcription factor NF-κB, which orchestrates the expression of pro-inflammatory genes during infections or tissue injury. However, NF-κB must be swiftly inactivated once the threat is resolved. This is where proteins like PDLIM2, an E3 ubiquitin ligase, come into play. PDLIM2 targets the p65 subunit of NF-κB for degradation, effectively silencing the inflammatory signal. Recent research by Sugimoto-Ishige et al. revealed that the F-box protein Fbxo16 is critical in this process. Fbxo16 enables PDLIM2 to interact with p65 within the nucleus. Without Fbxo16, p65 accumulates, prolonging cytokine production and increasing the risk of chronic inflammation (6).

Another important checkpoint is provided by IRAK3, a negative regulator of Toll-like receptor (TLR) and interleukin-1 receptor (IL-1R) signaling. Borghese et al. demonstrated that mice lacking IRAK3 exhibit more severe inflammatory arthritis. They produce higher levels of IL-1β and show reduced numbers of regulatory T cells, suggesting impaired immune tolerance. Moreover, IRAK3 expression is often diminished in autoimmune conditions such as SLE and inflammatory bowel disease, highlighting its relevance in human disease (7, 8).

In a different regulatory context, ARHGAP25, a Rho GTPase-activating protein, controls cytoskeletal dynamics and cell migration (9). Czárán et al. found that mice deficient in ARHGAP25 had significantly milder allergic contact dermatitis, associated with reduced immune cell migration and activation. This suggests that manipulating cytoskeletal signaling could represent a novel approach to treating chronic inflammation.

## 3 Innate immune cells and components in disease progression

Innate immune components are crucial regulators of inflammation. In JIA, Tang et al. reported an increased NET formation correlates with disease activity. Anti-TNF therapies reduce NET formation, indicating that NETs may serve as both biomarkers and therapeutic targets (10, 11). It has also been shown that the complement system, particularly the C5a-C5aR1 axis, is another potent driver of inflammation. An original article published by Vahldieck et al. indicated that in acute myocardial infarction, C5a disrupts the endothelial glycocalyx, reduces nitric oxide bioavailability, and recruits inflammatory cells, contributing to tissue injury. C5aR1 antagonists show therapeutic potential by preserving vascular integrity and reducing inflammation (12).

Besides these molecules, innate immune cells such as monocytes and macrophages play a key role in immune regulation. In the article by Akiyama et al., it has been shown that during hyperinflammatory conditions, such as cytokine release syndrome, monocytes undergo apoptosis to prevent overwhelming cytokine production. Disruption of this process, as shown in mouse models, leads to exaggerated immune responses and higher mortality. Maintaining monocyte homeostasis through controlled cell death or immunomodulation is emerging as a critical strategy in preventing systemic inflammatory damage (13, 14). Furthermore, Wang et al. identified that marked expression of Mincle receptors, transmembrane pattern recognition receptors on macrophages and neutrophils, exert pro-inflammatory and pro-fibrotic effects, involved in persistence of renal inflammatory microenvironment and accelerated renal fibrosis progression by inducing TNF production (15, 16).

## 4 Neutrophils in immunity and autoimmunity: balancing host defense and inflammation

Neutrophils are among the first responders in innate immunity, essential for eliminating pathogens. However, their prolonged activation can exacerbate inflammation, especially in sterile conditions. One key process is the formation of neutrophil extracellular traps (NETs), webs of DNA, histones, and granule proteins that capture microbes but also stimulate autoimmunity if dysregulated (10, 11). Li et al. reviewed the involvement of NETs in fibrotic and sterile inflammatory diseases. In conditions such as RA, NETs release modified self-antigens that provoke adaptive immune responses. Chen et al. showed that citrullinated proteins within NETs activate dendritic cells, encouraging T cell activation and the production of autoantibodies, thereby fueling the autoimmune cycle.

Given the dual nature of neutrophils, new strategies aim to reprogram rather than eliminate them. Raudszus et al. used cell-derived nanoparticles (CDNPs) to modulate neutrophil function. These CDNPs induced an anti-inflammatory phenotype, marked by increased IL-10 production and programmed cell death, facilitating inflammation resolution without compromising microbial defense. Such approaches reflect a growing interest in using nanomedicine to selectively steer immune cell function (17).

## 5 Inflammasomes and cytosolic DNA sensors as central drivers of chronic inflammation

Inflammasomes are multiprotein complexes that detect intracellular threats and activate caspase-1, leading to the release of IL-1β and IL-18 and triggering pyroptosis, a highly inflammatory form of cell death. The NLRP3 and AIM2 inflammasomes are the most studied and are increasingly implicated in chronic inflammatory diseases (18, 19). In idiopathic inflammatory myopathies, Sun et al. found that overactivation of NLRP3 and AIM2 correlates with disease severity. Inhibitors like MCC950, which specifically block NLRP3 activation, reduced inflammation in preclinical models and are being investigated in clinical trials (20, 21).

Parallel to inflammasomes, the cytosolic DNA sensing pathway, particularly cGAS-STING, is another major contributor to chronic inflammation. The article by Zhu and Zhou explained that while this pathway is vital in recognizing viral DNA, it can be aberrantly triggered by self-DNA released during cellular stress or apoptosis. The result is the chronic production of type I interferons and pro-inflammatory cytokines, a hallmark of diseases like SLE and dermatomyositis (22, 23). Small-molecule inhibitors of STING are currently under development, aiming to mitigate this persistent immune activation (24).

## 6 Metabolic and systemic influences on immune function

Beyond molecular signaling, immune function is tightly linked to systemic metabolic cues. Obesity, for instance, promotes chronic low-grade inflammation and can exacerbate inflammatory disorders. Shang and Zhao demonstrated that obesity impairs skin barrier integrity, alters the microbiome, and increases inflammatory mediators such as TNF-α and leptin. These changes worsen conditions like atopic dermatitis and reduce treatment efficacy (25, 26).

In contrast, nuclear receptors such as PPAR-γ serve as anti-inflammatory regulators. PPAR-γ activation inhibits pro-inflammatory gene transcription and promotes lipid metabolism, improving epithelial integrity and immune tolerance. Agonists targeting PPAR-γ have shown promise in reducing inflammation, particularly in obesity-related immune disorders (27, 28). This highlights the therapeutic potential of integrating metabolic interventions into inflammatory disease management.
